# Associations of body-related teasing with weight status, body image, and dieting behavior among Japanese adolescents

**DOI:** 10.15171/hpp.2017.15

**Published:** 2016-03-05

**Authors:** Naomi Chisuwa-Hayami, Toshi Haruki

**Affiliations:** Graduate School of Human Life Science, Osaka City University, Osaka, Japan

**Keywords:** Teasing, Bullying, Body image, Diet, Youth, Japanese

## Abstract

**Background:** Body-related teasing is known to be linked to body dissatisfaction and dieting behavior in adolescents. However, little is known about it in non-Western countries. This study aims to examine the prevalence of body-related teasing among Japanese adolescents and its connection to weight status, body image, and dieting behavior to consider implications for public health.

**Methods:** The design of this study is a cross-sectional study. An anonymous self-administrated survey was conducted with 1172 junior high school students in Higashi-Osaka City in Osaka Prefecture in Japan. The sampling method was non-random design. The survey items included self-reported height and weight, history and source of teasing, body image perception, and dieting behavior. A chi-square test and logistic regression analysis were used to examine the associations.

**Results:** A history of teasing was reported by 16.4% of boys and 32.5% of girls (P < 0.001, effect size = 0.19). The most common answer for source of teasing was friends (84.7% of boys’ teasing, 67.1% of girls’ teasing, P = 0.003, effect size = 0.19). Students who were overweight, of an upper-normal weight status, and perceived themselves as "fat" were at a greater risk of being teased. Additionally, students with a history of teasing were significantly likelier to display dieting behavior (odds ratios with confidence intervals: boys 4.06 [2.08–7.93], girls 2.40 [1.53– 3.75]).

**Conclusion:** Body-related teasing has a significant association with body image and dieting behavior in Japanese adolescents. A school-based education should be provided to reduce body-related teasing.

## Introduction

Comments and teasing about one’s body from others, including family and friends, is recognized as an influential factor in body image concerns and dieting behavior.^1^ In Western countries, body-related teasing is recognized as a form of stigma that contributes to obesity and may prevent people from maintaining a healthy behavior.^2,3^ A number of studies have been conducted to clarify the relationship between teasing and adolescents’ mental and physical health status as it pertains to obesity.^4,5^ The prevalence of teasing among adolescents was reported to be 23% in boys and 21% in girls in a large longitudinal study.^6^ These teasing experiences predicted binge eating behavior, with loss of control among boys and a frequent dieting behavior, defined as changing one’s eating patterns for weight loss, among girls in a 5-year follow-up.^6^ Another study with a racially diverse sample of sixth-grade children showed that the proportion of children being teased by peers was 40%, and those being teased by family was 36%. The children who had teasing experiences by peers or family showed a lower body satisfaction than those who did not have such experiences.^7^ Moreover, body-related teasing was associated with emotional wellbeing, such as low self-esteem and depression.^8^ It is right to refer to this as verbal bullying, and when directed at adolescents, it can drastically affect their mental health and wellbeing.

Although research on this topic has revealed the strong impact of such teasing, indicating the need for intervention to prevent teasing as a health promotion strategy, these studies were mostly conducted in developed Western countries. Little evidence has been reported on the correlation of teasing with body image and dieting behavior in non-Western countries.

Japan provides an interesting context in which to examine this issue. Along with economic development, Japan has adopted many Western lifestyle habits and trends. Similar health issues, such as an increase in obesity in adults, are prevalent in both the Western and Japanese contexts. However, according to the National School Health Survey in 2014, the prevalence of obesity among junior high school students in Japan was only 9.3% in boys and 7.8% in girls during that year.^9^ The prevalence of obesity among Japanese adolescents is quite low compared with that in Western countries, such as the US, where 20.3% of boys and 20.7% of girls in the same age range are obese.^10^ In Japan, although almost 85% of adolescents are classified to be of a normal weight status, they tend to be highly concerned about their body shape: 36.0% of boys and 79.0% of girls in junior high school and 36.9% of boys and 86.8% of girls in high school have expressed a desire to be thin.^11^ In addition, 27.0% of girls in junior high school and 40.4% of girls in high school have had dieting experiences.^11^ According to a study on female university students conducted by Maekawa,^12^ body-related teasing was associated with an increase in the importance of being thin and in body dissatisfaction. This result suggests that teasing can be an influential factor in Japanese adolescents’ body image, although most of these adolescents are of a normal weight status. However, a gap still exists in the literature in relation to the prevalence and sources of teasing among Japanese adolescents and the correlation of teasing with weight status, dieting behavior, and body image. Particularly, little is known about this issue among male Japanese adolescents. Understanding the impact of teasing is necessary to suggest appropriate school health education approaches for building a positive body image in line with the prevention of disordered eating behaviors and obesity. Therefore, this study aims to investigate the prevalence and sources of teasing and to examine the associations between teasing and body image and dieting behavior in a cross-sectional study of male and female Japanese adolescents.

## Materials and Methods

### Study design, participants, and sampling

This study used a cross-sectional study design. It was conducted as a part of the project, “Health and nutrition education for adolescents.” The sample size of this study was determined based on recommendation of Cohen^13^ and the previous study.^12^ Approximately 1000 participants were required to achieve a statistical power of 0.8 at α-level of 0.05 and detect a small effect size for the statistical analysis in this study. The participants were 1172 years 7 and 9 students recruited from four public junior high schools in Higashi-Osaka City (a large city) in Osaka Prefecture in Japan. The schools were selected on the basis of principals’ willingness to participate. The authors invited four public junior high schools from three areas in the city in May 2014, explained the study in detail, and then obtained permission with a written consent form from the principals in June 2014. A potential selection bias of the participants existed because of the non-random sampling method used. To reduce the selection bias within schools, all students in year 7 and 9 were recruited as the participants.

The participants in this study were general junior high school students. There was no outlier which needed to consider the inclusion or exclusion. Therefore, the criterion for data eligibility was determined by whether the participants provided answers to all questions on demographic characteristics and the dependent and independent variables. In accordance with this criterion, a total of 221 students were omitted because of missing data. The final dataset comprised 978 students (519 boys and 459 girls). The valid response rate was 83.4%.

### Procedure

A self-administered anonymous survey was conducted in the participating junior high schools in July 2014. Passive consent procedures were used, with the explanation that the participants had the right to decide whether to join, with no demerit if they decide otherwise. The homeroom teachers read out the survey instructions; the students then filled out the questionnaires during homeroom, and these were collected thereafter.

### Measures

Degree of obesity

Based on self-reported height and weight, the degree of obesity used in the Annual School Health Survey of Japan was calculated to evaluate weight status, with age and gender adjusted to fit the case of children and adolescents.^9^ Weight status is usually categorized into the following three groups: underweight (under −20% of the degree of obesity), normal (−20% to +20%), and obese (over +20%). In general terms, most students were of a normal weight, so the normal category was further divided into three groups according to tertile: lower, middle, and upper. The resulting five weight statuses were then used for analysis.

*History of teasing*

History of teasing was measured by a dichotomous question based on perceived teasing. Students who reported such a history were asked to identify their source of teasing (friends, family, or others) and thus allow multiple selections.

Body image and dieting behavior

Body image was measured by the question, “How do you describe your body shape?” There were three possible answers: thin, normal, and fat. Dieting behavior was measured by the question, “Have you ever gone on a diet to lose weight?” There were four possible answers: “I did dieting under the supervision of a doctor or professional,” “I did dieting to lose weight,” “I thought to try, but I have not done it,” and “Never.” In this study, dieting behavior was defined as “any efforts to lose weight or to be thinner.” Students who answered “I thought to try, but I have not done it” and “Never” were placed into the same category as those who answered “No” to classify the students into two groups (“Yes” and “No”).

Covariates

Previous research has shown that weight status is a significant factor in body image and disordered eating behaviors.^14,15^ It is also well demonstrated that distorted body image and dieting behaviors increases as one’s grade in school advanced.^16^ Both degree of obesity and grade were therefore included as covariates to control the possible effects from these variables.

### Statistical analysis

SPSS statistics for Windows version 23.0 (IBM Inc., Armonk, NY, USA) was used for the entire analysis. First, descriptive analyses were conducted to obtain the prevalence of each item. A chi-square test was then performed to examine gender differences. If the expectation for more than two cells is less than five, Fisher extract test was used. Significant associations were found between teasing and gender, as well as between weight status and gender. Gender was considered as a cofounder, so we stratified the participants by gender to adjust the influence.

Second, unadjusted associations between teasing and weight status and body image were examined with a chi-square test or Fisher extract test. Multivariate logistic regression with forward selection was then performed to examine the associations between weight status and teasing while adjusting for grade.In this analysis, weight status was a primary study factor, and teasing was a primary outcome. The mediation effect of body image was examined by addition of body image to the regression model to consider the changes in association between weight status and teasing (Model 1).

Finally, associations between teasing and dieting behavior were examined. Unadjusted associations were analyzed with a chi-square test or Fisher extract test. Multivariate logistic regression with forward selection was then performed to examine the associations, with history of teasing and dieting behavior adjusted for grade and degree of obesity as covariates (Model 1). In this analysis, teasing was a primary study factor, and dieting behavior was a primary outcome.

The effect size (ES) of a chi-square test or Fisher extract test was shown as φ coefficients or Cramer’s* V*. The statistical significance was set at 0.05.

## Results

### Demographic characteristics of the participants

The demographic characteristics of the participants are shown in Table 1. Of the participants, 53.1% were males, and the rest of them (46.9%) were females. The mean age of the study sample was 13.2 (SD: 1.08) years. Regarding grade, 53.4 % of the participants were in year 7, and 46.6% were in year 9.

### Descriptive features of history of teasing by gender

The prevalence of history and source of teasing by gender is shown in Table 2. In total, 16.4% of boys and 32.5% of girls reported being teased. A history of teasing was more common in girls than in boys (*P*<0.001, ES=0.19). The percentage of teasing by friends in the total teasing history for each gender was significantly higher in boys (84.7%) than in girls (67.1%) (*P*=0.003, ES=0.19). The percentage of teasing by family in the total teasing history showed different results. Girls who were teased by family showed a higher percentage (62.4%) than boys (38.8%) (*P*<0.001, ES=0.23).

### Prevalence of history of teasing by weight status and body image by gender

The chi-square test showed a significant association between history of teasing and weight status in both boys and girls (Table 3). Among boys, history of teasing in total (*P*<0.001, ES=0.21) and history of teasing by friends (*P*=0.002, ES=0.19) had significant associations with weight status. Boys in the overweight category and in the upper-normal weight category reported a higher prevalence of teasing in total (38.5%, 20.8%) and by friends (33.3%, 17.6%). Among girls, history of teasing in total (*P*<0.001, ES=0.35), by friends (*P*<0.001, ES=0.28), and by family (*P*<0.001, ES=0.28) had significant associations with weight status. Similar to boys, girls in the overweight and upper-normal weight categories reported more teasing than those in the lower weight categories.

Associations between a history of teasing and body image were confirmed in both boys and girls with a chi-square test (Table 3). Among boys, significant differences were found in all sources of teasing by body image: in total (*P*<0.001, ES=0.22), by friends (*P*<0.001, ES=0.24), and by family (*P*=0.023, ES=0.13). Boys who perceived themselves as “fat” reported a higher prevalence of a history of teasing in total (35.1%), by friends (33.8%), and by family (13.5%). Among girls, significant differences were found in all sources of teasing (*P*<0.001, ES=0.40, 0.31, 0.30 respectively). Girls who perceived themselves as “fat” showed a higher prevalence of a history of teasing in total (52.0%), by friends (33.0%), and by family (34.0%).

### Associations of history of teasing and weight status by gender

Table 4 shows the results of logistic regression analysis. Grade was used as a covariate, however, it was not significant in this analysis. Logistic regression analysis showed that boys and girls who were overweight and of upper-normal weight were at a greater risk of being teased than those who were underweight or of lower- or middle-normal weight, as shown in Crude (ORs [CIs] of overweight: boys 5.51 [2.41-12.59[, girls 6.22 [2.70-14.31]), (ORs [CIs] of the upper-normal weight: boys 2.31 [1.21-4.39], girls 3.31 [1.95-5.60]). In addition, the analysis similarly indicated that boys and girls who perceived themselves as “fat” were also at a greater risk of being teased (ORs [CIs]: boys 4.14 [2.29-7.47], girls 6.53 [4.12-10.36]). Further analysis, including two items together as shown in Model 1, showed decreased ORs with CIs in all weight categories except the upper-normal weight in boys and “fat” as a body image; however, the OR with CIs of “thin” as a body image increased in both boys (ORs [CIs]: 1.58 [0.90-2.79] to 2.24 [1.15–4.37]) and girls (ORs [CIs]: 4.82 [2.08–11.22] to 6.48 [2.53–16.61]).

### Associations of dieting behavior with history of teasing by gender

No students reported that they had dieted under the supervision of a doctor or professional. Students who reported being teased showed a significantly higher percentage of dieting behavior (boys: 25.9%, girls: 46.3%) than those who did not (boys: 6.0%, girls: 23.2%) (boys: *P*<0.001, ES=0.25, girls: *P*<0.001, ES=0.23) (Table 5). Similarly, a history of teasing by friends or family also showed significant associations with dieting behavior.

Adjusted analysis for grade and degree of obesity returned similar results, as shown in Model 1 in Table 6. Regardless of the type of teasing, both boys and girls who reported being teased were at a greater risk of adverse dieting behavior. Among boys, the ORs (CIs) for this behavior increased by history of teasing in total (OR [CI]: 4.06 [2.08–7.93]), by friends (OR [CI]: 3.62 [1.81–7.23]), and by family (OR [CI]: 3.67 [1.52–8.86]). Similarly, among girls, the ORs (CIs) for this behavior increased by history of teasing in total (OR [CI]: 2.40 [1.53–3.75]), by friends (OR [CI]: 1.93 [1.18–3.14]), and by family (OR [CI]: 2.02 [1.23–3.33]).

## Discussion

The findings in this study are mostly consistent with those of previous studies that focused on the association between teasing and weight status conducted in Western countries.^6,14^ Although fewer students of overweight status can be found in Japan than in Western countries, clear associations exist between weight status and history of teasing among Japanese adolescents. Even non-overweight adolescents can be teased if they are bigger than others, which may result in unnecessary dieting behavior. Regarding prevalence, about 25% of adolescents in a study by Haines et al^17^ and about 30% in that by Brixval et al^18^ reported being teased. With the prevalence of teasing in these previous studies considered, the results of the current research seem to be consistent with such prevalence, particularly in girls. Japanese adolescent boys may have lower prevalence of teasing as boys also reported a similar prevalence as girls in previous studies. However, because of different measures, the results cannot be simply compared directly.

Friends were the most common sources of teasing in both boys and girls. This finding implies that teasing regularly occurs within a group of friends or classmates as a part of everyday life. In girls, family was also common source of teasing. Keery et al indicated that teasing by parents is a predictor of body dissatisfaction, restricted eating, low self-esteem, and depression.^19^ This teasing from one’s family may compromise the safety of the home environment for adolescents, who are in a critical period of developing their self-esteem. A review by Menzel et al^20^ reported that the results of previous studies on sources of teasing were inconsistent, and determining which factor had a stronger impact was impossible. In broad terms, both friends and family are strong, influential factors. Social comparison theory, propounded by Festinger,^21^ states that people usually compare themselves not with an objective standard but with others. Therefore, adolescents may not refer to the degree of obesity, which is the absolute standard. Most Japanese adolescents are of a normal weight status, so boys and girls in the relatively larger group are inevitably the targets of teasing. In addition, students who perceive themselves as “fat” are also at a greater risk of being teased. Body image mediated the associations between weight status and teasing. Those who perceived themselves as “thin” also showed a high risk of being teased. Boys and girls who have a “negative” body image may be vulnerable to teasing, and they may perceive it to a greater degree than those who do not have a negative body image.^6^

Associations between a history of teasing and dieting behavior were confirmed in both boys and girls, as reported in previous studies.^4,6,14^ In girls, nearly half who had a history of teasing showed such a behavior. However, the risk of dieting based on teasing was higher in boys than in girls. The results indicates that girls are influenced by different factors which may result in an unhealthy dieting behavior. As reported previously, teasing causes body dissatisfaction, which is a strong motivator of such a behavior.^12^ A pathway by which teasing leads directly to heavier dieting may also exist. The results imply that teasing can be a trigger that eventually causes adverse dieting behavior.

### Limitations

This study has some limitations which reduce the generalizability of the study results. First, the sampling method was non-random sampling, so a potential selection could have been involved. However, the sample was relatively large and selected from three different areas which covered the major areas of the city. The results should be useful in developing a health education program in this region. Second, the history of teasing and dieting behaviors were assessed by a single question. This may limit the validity and reliability of the data interpretation. For instance, we did not consider in the questions the frequency of teasing and exactly when it occurred. This study also did not measure the level of teasing because it may broadly range from making fun to severe bullying. Definition of dieting behaviors in this study was quite broad. Dieting methods could not be clarified. The measurement of teasing and dieting behaviors would be useful to examine the level of them. Third, weight status was calculated on the basis of self-reported height and weight. However, the data for height, weight, and weight status in this study were similar to those in the Annual National School Health Survey in 2014.^9^ Finally, the cross-sectional design of this study could not clarify the causes and effects of teasing. Longitudinal studies should be conducted in the future to examine the effect of teasing in greater detail.

### Implications for practice

The findings of the current study indicate a need for intervention to prevent dieting behavior and build a positive body image among Japanese adolescents by reducing teasing. Three levels of possible approaches could be implemented. The first is the school-level approach. Boys and girls are commonly teased by their friends. A school-based educational program should be provided so that students could avoid body-related teasing from friends and to promote positive talk about their bodies. The second is the family-level approach, which would be effective particularly for girls. Health professionals should raise awareness on the negative effect of teasing at home among parents and siblings. Finally, an individual approach to build a positive body image would be necessary. Most adolescents are not in the overweight category. Therefore, health educators should provide these adolescents with opportunities to increase acceptance of the diversity of body shapes and to build a positive body image so that the incidence of teasing and adverse unhealthy eating behavior is reduced. Haines et al reported that a program, V.I.K. (Very Important Kids), which included an after-school program and incorporated both school environment and family components, reduced the percentage of students who reported being teased compared with those in the control group.^22^ This program did not just focus on overweight students; the effect was also seen in students of a normal weight status. A school-based comprehensive program may contribute to the reduction of weight-related issues among Japanese adolescents.

## Conclusion

Boys and girls who are overweight and perceive themselves as “fat” are at a greater risk of being teased, and the same is the case for those who are not overweight but of an upper-normal weight status. Body image mediates this association. A history of teasing is associated with dieting behavior among both boys and girls. Health educators should provide a school-based program that will increase acceptance of the diversity of body shapes and reduce teasing among Japanese adolescents so that healthy eating behavior and a positive body image are promoted.

## Ethical approval

All the procedures in this study were reviewed and approved by the Human Ethics Committee of the Graduate School of Human Life Science (Ethics code: 14-10), as the survey included personal and sensitive questions.

## Competing interests

We declare no competing interest.

## Authors’ contributions

NC designed, managed and analysed this study and prepared the manuscript. TH was involved in the conception of the study and assisted revision.

## Acknowledgments

We would like to thank all individuals who participated in this study, including all teachers who helped with the survey. We would also like to thank Professor Kohta Suzuki for the assistance in the statistical analysis. No funding was used to conduct this study.

**Table 1:** Demographic characteristics of study population in total and by gender

**Variable**	**Total (n=978)**		**Boys (n=519)**		**Girls (n=459)**	
	**n**	**%**	**n**	**%**	**n**	**%**
Gender						
Male	519	53.1	-	-	-	-
Female	459	46.9	-	-	-	-
Grade						
Year 7	522	53.4	276	53.2	246	53.6
Year 9	456	46.6	243	46.8	213	46.4
Age (years)^a^	13.2 (1.08)		13.2 (1.09)		13.2 (1.08)	
Degree of obesity^a^	-2.69 (13.59)		-1.61 (13.58)		-3.91 (13.51)	

^a^Mean (SD) is presented for age and degree of obesity.

**Table 2:** Prevalence of history of teasing and source of teasing by gender

**Variable**	**Total (n = 978)**		**Boys (n = 519)**		**Girls (n = 459)**		***P *** **value** ^a^	**ES** ^b^
	**n**	**%**	**n**	**%**	**n**	**%**		
History of teasing								
Yes	234	23.9	85	16.4	149	32.5	<0.001	0.19
No	744	76.1	434	83.6	310	67.5		
Source of teasing^c^								
Friends	172	73.5	72	84.7	100	67.1	0.003	0.19
Family	126	53.8	33	38.8	93	62.4	<0.001	0.23
Others	18	7.7	3	3.5	15	10.1	0.071	0.12

^a^ *P* values represent a chi-square test. ^b^ ES: effect size (φ) ^c^ Percentage of source of teasing shows the ratio of total history of teasing in each gender.

**Table 3:** Prevalence of history of teasing by weight status and body image by gender

	**Total**				**Friends**				**Family**			
	**n**	**%**	***P*** **value** ^a^	**ES** ^b^	**n**	**%**	***P*** **value** ^a^	**ES** ^b^	**n**	**%**	***P*** **value** ^a^	**ES** ^b^
**Boys**												
Weight status												
Underweight (n=10)	1	10.0	<0.001	0.21	1	10.0	0.002	0.19	0	0.0	0.074	0.13
Lower normal weight (n=154)	20	13.0			13	8.3			8	3.9		
Middle normal weight (n=157)	16	10.2			19	12.3			6	5.1		
Upper normal weight (n=159)	33	20.8			28	17.6			14	8.8		
Overweight (n=39)	15	38.5			13	33.3			6	15.4		
Body image												
Thin (n=134)	23	17.2	<0.001	0.22	20	14.9	<0.001	0.24	10	7.5	0.023	0.13
Normal (n=311)	36	11.6			29	9.3			14	4.5		
Fat (n=74)	26	35.1			25	33.8			10	13.5		
**Girls**												
Weight status												
Underweight (n=27)	7	25.9	<0.001	0.35	7	25.9	<0.001	0.28	2	7.4	<0.001	0.28
Lower normal weight (n=132)	23	17.0			18	13.6			17	12.9		
Middle normal weight (n=135)	31	23.5			18	13.3			16	11.9		
Upper normal weight (n=133)	67	50.4			40	30.1			47	35.3		
Overweight (n=32)	21	65.6			17	53.1			11	34.4		
Body image												
Thin (n=27)	12	44.4	<0.001	0.40	12	44.4	<0.001	0.31	4	14.8	<0.001	0.30
Normal (n=232)	33	14.2			22	9.5			21	9.1		
Fat (n=200)	104	52.0			66	33.0			68	34.0		

^a^ *P* values represent a chi-square test or Fisher extract test. ^b^ ES: Effect size (Cramer’s V)

**Table 4:** Logistic regression analysis for history of teasing by weight status and body image by gender (OR [95% CI])

	**Crude**			**Model 1**		
	**OR**	**CI**	***P*** **value** ^a^	**OR**	**CI**	***P*** **value** ^a^
**Boys**						
Weight status						
Underweight (n=10)	0.98	(0.12-8.24)	0.99	0.77	(0.089-6.68)	0.81
Lower normal weight (n=154)	0.44	(0.65-2.65)	0.44	1.06	(0.51-2.23)	0.87
Middle normal weight (n=157)	1.00	-	<0.001	1.00	-	0.047
Upper normal weight (n=159)	2.31	(1.21-4.39)	0.011	2.36	(1.20-4.64)	0.013
Overweight (n=39)	5.51	(2.41-12.59)	<0.001	3.44	(1.29-9.21)	0.014
Body image						
Thin (n=134)	1.58	(0.90-2.79)	0.113	2.24	(1.15-4.37)	0.018
Normal (n=311)	1.00	-	<0.001	1.00	-	0.004
Fat (n=74)	4.14	(2.29-7.47)	<0.001	2.65	(1.32-5.31)	0.006
* Grade*	-	-	0.96	-	-	0.82
**Girls**						
Weight status						
Underweight (n=27)	1.14	(0.44-2.95)	0.79	0.84	(0.27-2.64)	0.77
Lower normal weight (n=132)	0.67	(0.37-1.22)	0.19	0.74	(0.38-1.45)	0.38
Middle normal weight (n=135)	1.00	-	<0.001	1.00	-	-
Upper normal weight (n=133)	3.31	(1.95-5.60)	<0.001	2.36	(1.35-4.13)	0.003
Overweight (n=32)	6.22	(2.70-14.31)	<0.001	3.42	(1.43-8.18)	0.006
Body image						
Thin (n=27)	4.82	(2.08-11.22)	<0.001	6.48	(2.53-16.61)	<0.001
Normal (n=232)	1.00	-	<0.001	1.00	-	<0.001
Fat (n=200)	6.53	(4.12-10.36)	<0.001	3.96	(2.36-6.63)	<0.001
* Grade*	-	-	0.57	-	-	0.96

Abbreviaton: OR, odds ratio. ^a^ *P* values represent test for association in logistic regression analysis. Note: Variables in italics are covariates.

**Table 5:** Prevalence of dieting behaviors by history of teasing by gender

	**Dieting behaviors**										
	**Boys**					**Girls**				
	**N**	**n**	**%**	***P*** **value** ^a^	**ES** ^b^	**N** ^c^	**n** ^d^	**%**	***P*** **value** ^a^	**ES** ^b^
History of teasing										
Total										
Yes	85	22	25.9	<0.001	0.25	149	69	46.3	<0.001	0.23
No	434	26	6.0			310	72	23.2		
Friends										
Yes	74	19	25.7	<0.001	0.23	100	46	46.0	<0.001	0.18
No	445	29	6.5			359	95	26.5		
Family										
Yes	34	10	29.4	<0.001	0.18	93	45	48.4	<0.001	0.19
No	485	38	7.8			366	96	26.2		

^a^ *P* values represent a chi-square test or Fisher extract test. ^b^ ES: Effect size (φ). ^c^ N represents number reporting teasing (yes or no). ^d^ n represents number reporting dieting behaviors.

**Table 6:** Logistic regression analysis for dieting behaviors by history of teasing by gender [OR (95% CI)]

	**Crude**			**Model 1**		
	**OR**	**CI**	***P*** **value** ^a^	**OR**	**CI**	***P*** **value** ^a^
**Boys (n = 549)**						
Total history of teasing (yes)	5.48	(2.93-10.26)	<0.001	4.06	(2.08-7.93)	<0.001
* Grade *	-	-	0.093	2.20	(1.13-4.29)	0.021
* Degree of obesity*	1.34	(1.21-1.48)	<0.001	1.05	(1.03-1.07)	<0.001
Teasing by friends (yes)	4.96	(2.61-9.43)	<0.001	3.62	(1.81-7.23)	<0.001
* Grade *	-	-	0.093	-	-	0.15
* Degree of obesity*	1.34	(1.21-1.48)	<0.001	1.17	(1.16-1.43)	<0.001
Teasing by family (yes)	4.90	(2.18-11.00)	<0.001	3.67	(1.52-8.86)	0.0
* Grade *	-	-	0.093	-	-	0.11
* Degree of obesity*	1.34	(1.21-1.48)	<0.001	1.32	(1.19-1.46)	<0.001
**Girls (n = 459)**						
Total history of teasing (yes)	2.85	(1.88-4.32)	<0.001	2.40	(1.53-3.75)	<0.001
* Grade *	2.16	(1.44-3.23)	<0.001	2.31	(1.51-3.52)	<0.001
* Degree of obesity*	1.20	(1.11-1.30)	<0.001	1.02	(1.01-1.04)	0.01
Teasing by friends (yes)	2.37	(1.50-3.74)	<0.001	1.93	(1.18-3.14)	0.008
* Grade *	2.16	(1.44-3.23)	<0.001	1.95	(1.28-2.96)	0.002
* Degree of obesity*	1.20	(1.11-1.30)	<0.001	1.16	(1.07-1.25)	<0.001
Teasing by family (yes)	2.64	(1.65-4.21)	<0.001	2.02	(1.23-3.33)	0.006
* Grade *	2.16	(1.44-3.23)	<0.001	1.89	(1.24-2.88)	0.003
* Degree of obesity*	1.20	(1.11-1.30)	<0.001	1.15	(1.23-3.33)	0.001

Abbreviaton: OR, odds ratio. ^a^ *P* values represent test for association in logistic regression analysis. Note: Variables in italics are covariates.
